# Sustainable Fully Inkjet-Printed Humidity Sensor Based on Ionic Liquid and Hydroxypropyl Cellulose

**DOI:** 10.1021/acsami.5c00505

**Published:** 2025-05-25

**Authors:** Mikel Rincón-Iglesias, Peter Krebsbach, Daniela M. Correia, Cristian Mendes-Felipe, Senentxu Lanceros-Méndez, Gerardo Hernandez-Sosa

**Affiliations:** † BCMaterials, Basque Center for Materials, Bldg. Martina Casiano, UPV/EHU Science Park Barrio Sarriena s/n, 48940 Leioa, Spain; ‡ Light Technology Institute, 150232 Karlsruhe Institute of Technology, Engesserstr. 13, 76131 Karlsruhe, Germany; § InnovationLab, Speyerer Straße 4, 69115 Heidelberg, Germany; ∥ Centre of Chemistry, 56059 University of Minho, 4710-057 Braga, Portugal; ⊥ Ikerbasque, Basque Foundation for Science, Plaza Euskadi 5, 48009 Bilbao, Spain; # Institute of Microstructure Technology, Karlsruhe Institute of Technology, 76344 Eggenstein-Leopoldshafen, Germany

**Keywords:** Inkjet Printing, Sensors, Humidity, Cellulose, Ionic Liquid

## Abstract

The increasing number of sensors contributing to the Internet of Things (IoT) aggravates the e-waste generated globally. Thus, it is an urgent necessity to develop more sustainable sensors. This paper presents a fully inkjet-printed dual-response (electrical and visual) humidity sensor based on hydroxypropyl cellulose (HPC) and the ionic liquid bis­(1-butyl-3-methylimidazolium) tetrachloronickelate ([Bmim]_2_[NiCl_4_]). The active layer was printed on interdigitated silver electrodes on a flexible cellulose acetate substrate. The optimized ink includes HPC, [Bmim]_2_[NiCl_4_], ethylene glycol, water, and Tergitol. HPC and the IL exhibit excellent compatibility, forming homogeneous films without phase separation even at high IL concentration. The printed sensor for an IL content of 50 wt % demonstrates a proportional response when varying the relative humidity (RH) from 30 to 90 RH%, with a high sensitivity of 163, comparable to that of a commercial reference sensor, a low hysteresis of 1.5 RH%, and a fast response time of 0.8 s. In addition, a visual response from colorless to cyan is observed upon dehydration. This color change is visible to the naked eye for a relative humidity below 30 RH% when a transmittance lower than 93% is obtained in the visible spectra. This dual-response humidity sensor, fabricated from sustainable materials and low-cost printing technology, has great potential for a variety of applications, including environmental monitoring, smart agriculture, fire safety, and quality control in the food industry.

## Graphical abstract

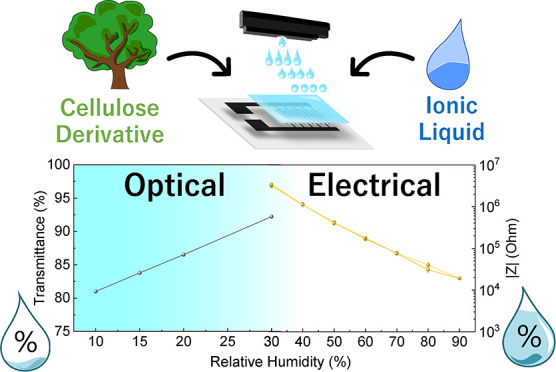

## 1 Introduction

Humidity sensors have gained relevance in the scope of the Internet of Things (IoT) scenario, as the ubiquitous humidity level is a key factor in various fields including environmental monitoring, smart agriculture, the food industry, and packaging.^1,2^ Advances in printed electronics boost the fabrication of flexible, lightweight, efficient, customizable, and low-cost sensors. Consequently, the application areas of humidity sensors are being extended to wearables, healthcare, robotics, and human–machine interaction.^3^

In printed humidity sensors, carbon-based materials, polymer composites, and ceramic materials are widely used as humidity-sensing materials.^4,5^ In the framework of flexible and transparent electronics contributing to the IoT, the active layer is typically deposited onto polyimide (PI), polyethylene terephthalate (PET), or polyester sheets.^6^ The use of these nondegradable materials entails a high amount of waste, coined as e-waste, and aggravates the current problem of plastic accumulation. In 2022, a record of total e-waste was reached, generating 62 billion kg, of which only 22% were formally collected and recycled.^7,8^ Taking into account that part of the e-waste are short service-life devices or even single-use,^9^ there is an urgent need to develop sensors based on not only recyclable but also biodegradable materials processed with resource-efficient printing technologies for portable use.

To reduce the environmental footprint, fossil-sourced polymers can be substituted with naturally derived biopolymers. Among the various natural-sourced polymers, cellulose emerges as a versatile material that can be employed as a substrate, matrix, or filler in a printed electronic component. Cellulose is the most abundant biomaterial in the biosphere and is characterized by high strength, stiffness, biocompatibility, biodegradability, low cost, and hydrophilicity. Thus, cellulosic paper or cellulose nanofibers (CNFs) have been employed as substrates for the development of printed humidity sensors.^10^ In addition, the reactions of its hydroxyl groups allow to tailor the physicochemical properties of the polysaccharide.^11^ The substitution of hydroxyl groups with acetyl groups results in cellulose acetate (CA), the most widely consumed cellulose derivative in the world. CA has been extensively used in packaging, films, or nanofiber production due to its transparency, thermoplastic behavior, biodegradability, low cost, good chemical and mechanical stability, and excellent film-forming capacity.^12–15^ Accordingly, it is employed as a sustainable colorless substrate in printed electronics.^16^ On the other hand, the modification of −OH groups in cellulose avoids the formation of strong inter- and intrahydrogen bonding leading to water-soluble cellulose derivatives, where carboxymethyl cellulose (NaCMC), methylcellulose (MC), hydroxyethyl cellulose (HEC), and hydroxypropyl cellulose (HPC) are some of the most employed ones. Among them, HPC stands out for its excellent film-forming capacity, high transparency, resistance to oils, and dispersibility of fillers. Thus, it has been extensively used as a matrix or binder in composites for developing smart materials.^17,18^

Usually, the functionality of the inks comes from micro- and nanoparticulate functional fillers in a composite material. Nevertheless, the low stability of some particles in solution and the aggregation can limit the printability by inkjet printing or entangle the composition of inks.^19^ To overcome these limitations, inks based on functional ionic liquids (ILs), which are liquid electrolytes with low melting temperatures (<100 °C), can present significant advantages for the printing process.^20^ The liquid nature of ILs means they do not form aggregates, and their high boiling point slows the drying process, both of which help to reduce clogging during the printing process and therefore can extend the life of replaceable components.^21^ In addition, the combination of IL and HPC avoids annealing or energy-consuming postprocessing after printing. The ILs provide the pursued response such as capacitive, conductive, magnetic, luminescent, or chromic among others with water solubility, nonvolatility, and chemical and electrochemical stability.^22^ Among the ILs, imidazolium-based ILs are widely used due to their ease of synthesis and versatility as cationic frameworks for ILs. However, the aromatic ring presents toxicity, which can lead to environmental risks when it is disposed of in nature.

The printability of water-soluble cellulose derivatives has been commonly performed by direct ink writing (DIW) and screen-printing techniques, but their use in inkjet printing (IJP) fabrication has been scarcely reported.^23–25^ IJP is one of the most developed techniques to print functional materials, as it combines high-resolution patterns and scalability with reduced material consumption and waste. This noncontact printing process is carried out at low temperatures where only the ink that covers the digital pattern is deposited.^26,27^

In this work, we fully inkjet printed a more sustainable humidity sensor with dual responses: electrical and optical. The active layer of the sensor is based on hydroxypropyl cellulose and the IL bis­(1-butyl-3-methylimidazolium) tetrachloronickelate ([Bmim]_2_[NiCl_4_]). First, the material properties were evaluated and showed excellent compatibility, forming homogeneous layers even at 50 wt % of IL. Apart from the high conductivity, this IL presents a color change dependent on the humidity level, turning cyan when dehydrated and colorless in the hydrated state. Impedance moduli of sensors were measured for a wide range of relative humidity (RH), from 30 to 90 RH%.

The performance of drop-cast and inkjet-printed sensors was evaluated. Printed sensors performed a linear response (log scale) and a comparable sensitivity with the sensitivity obtained for the commercial reference. The cyan color of the IL can be observed below 30 RH% for the printed sensors, and the color intensity increases linearly for lower humidity. Interestingly, this visual detection at lower humidity than 30 RH% matches perfectly with the extended rule of thumb of “30–30–30” for preventing extreme wildfire potential (ambient temperature higher than 30 °C, relative humidity lower than 30 RH%, and wind speed higher than 30 km h^–1^).^28,29^

## 2 Materials and Methods

### 2.1 Materials

HPC, powder (*M*_w_ ≈ 100,000 g·mol^–1^), silver nanoink (Silverjet DGP-40LT-15C), ethylene glycol (≥99%), and Tergitol 15-S-9 (T15S9, 30 mN/m, Dow) were purchased from Sigma-Aldrich. CA with a thickness of 180 μm was supplied by Rachow Kunststoff-Folien GmbH. The [Bmim]_2_[NiCl_4_], 99% was synthesized as previously reported.^30–32^

### 2.2 Free-Standing Film Preparation

Freestanding films of ∼40 μm comprising [Bmim]_2_[NiCl_4_] in HPC from 0 to 50 wt % were prepared to evaluate material properties. For this purpose, HPC was dissolved in water at a concentration of 10 wt/vol %. Then, the appropriate quantity of IL corresponding to 10, 20, 30, 40, and 50 wt % was incorporated into the solution, which was subsequently blade-coated onto a glass substrate and dried at room temperature (RT).

### 2.3 Inkjet Printing

The HPC/IL solution was adapted to be printed by an inkjet. Therefore, the final HPC concentration was reduced until 5 mg·mL^–1^, and ethylene glycol (EG) was also added to the solution in a volume ratio of H_2_O:EG = 1:2. Finally, Tergitol was used as a surfactant in 0.03 vol % of the solution (2.9 wt % with respect to HPC/IL). Sensors were fully printed onto CA flexible substrates. All the inks were printed with a Samba Cartridge printhead by Fujifilm, a multijet cartridge of 12 nozzles ejecting 2.4 pL. The CA substrate was first cleaned with isopropanol and cleanroom wipes to remove the possible residues of oils and fats. Then, it was further cleaned with Ar plasma (Diener NANO Plasma Cleaner) at a flow rate of 65 mL·min^–1^ for 5 min with a pressure of 0.35 mbar.

The first layer consists of a silver interdigitated electrode (IDE), printed at 1100 dpi with a PiXDRO LP50 by Süss MicroTec. After the printing, the pattern was annealed at 100 °C for 30 min on a hot plate (Präzitherm). Then, the HPC/IL-based ink layers were printed with a Dimatix printer (DMP 2831) at a resolution of 2450 dpi completely covering the digits of the IDE. To accelerate the drying process, the prints were vacuum-dried at 35 °C and 700 mbar in an oven. A maximum of 10 layers of HPC/IL were printed for a sensor, where a maximum of three layers were printed nonstop before drying conditions.

### 2.4 Sample Characterization

#### 2.4.1 Ink Characterization

The viscosities of solutions were measured with an m-VROC Viscometer (RheoSense). The surface tension of inks, the surface free energy of substrates, and the contact angle between solutions and substrates were measured with a KRÜSS DSA100 drop shape analyzing system.

#### 2.4.2 Film and Print Characterization

Optical images of the films and printings were taken with a Canon 6D Camera. For higher resolution images, optical micrographs were taken with an Eclipse 80i microscope by Nikon, and a Hitachi S-4800 field emission scanning electron microscope (FESEM) at an acceleration voltage of 5 kV was also used. In addition, Ni mapping analyses were performed with scanning electron microscopy (SEM) coupled with energy-dispersive X-ray spectroscopy (EDX) on a DSM 982 Gemini instrument (Zeiss). A stylus profilometer (Dektak 150, Bruker) was used to determine the thickness of the printed layers. Measurements of color change were performed with an ultraviolet–visible (UV–vis) AvaSpec ULS3648 spectrophotometer equipped with an AvaLight-DHS-Bal light source, both by Avantes. Fourier transform infrared (FTIR) spectroscopy measurements in attenuated total reflection (ATR) mode were carried out on a Jasco FT/IR-6100 spectrometer equipped with diamond ATR optics. For FT/IR spectra, 64 scans were recorded in the range 3800–600 cm^–1^ with a resolution of 4 cm^–1^.

#### 2.4.3 Sensor Characterization

Electrical and optical measurements for the humidity sensors were performed in a climate chamber (MKF115, Binder GmbH) to control the temperature and relative humidity (RH). The humidity ranges are between 25–95 RH% for temperatures below 40 °C. For temperatures above 40 °C, the range increases to 10–90 RH%. Electrochemical impedance spectroscopy (EIS) was carried out with an Autolab potentiostat/galvanostat (Autolab PGSTAT302N, Metrohm) in a range of frequencies 10^–1^–10^6^ Hz with a 0.3 *V*_rms_ sine wave, over at least three and a maximum of six humidity cycles, where a cycle means 30 → 90 → 30 RH%. The results were compared to the commercial electrolytic humidity sensor EFS-10 (B+B Thermo-Technik GmbH), used as a reference. Details on the long-term and detection speed characterization can be found in the Supporting Information.

## 3 Results and Discussion

The sensor’s architecture consists of two layers on a CA substrate. First, a conductive IDE pattern was printed, followed by printing an active layer onto the IDE in a square shape. The fabrication process and performance of the sensors are schematically described in Figure 1, including the composition of the ink for printing the active layer and the postprocessing. Then, this sensor fabrication only needs two inks: (i) Ag ink for printing the electrodes and (ii) a formulated green solvent-based ink for the active layer composed of HPC and [Bmim]_2_[NiCl_4_] at a concentration of 50 wt %.

**Figure 1:**
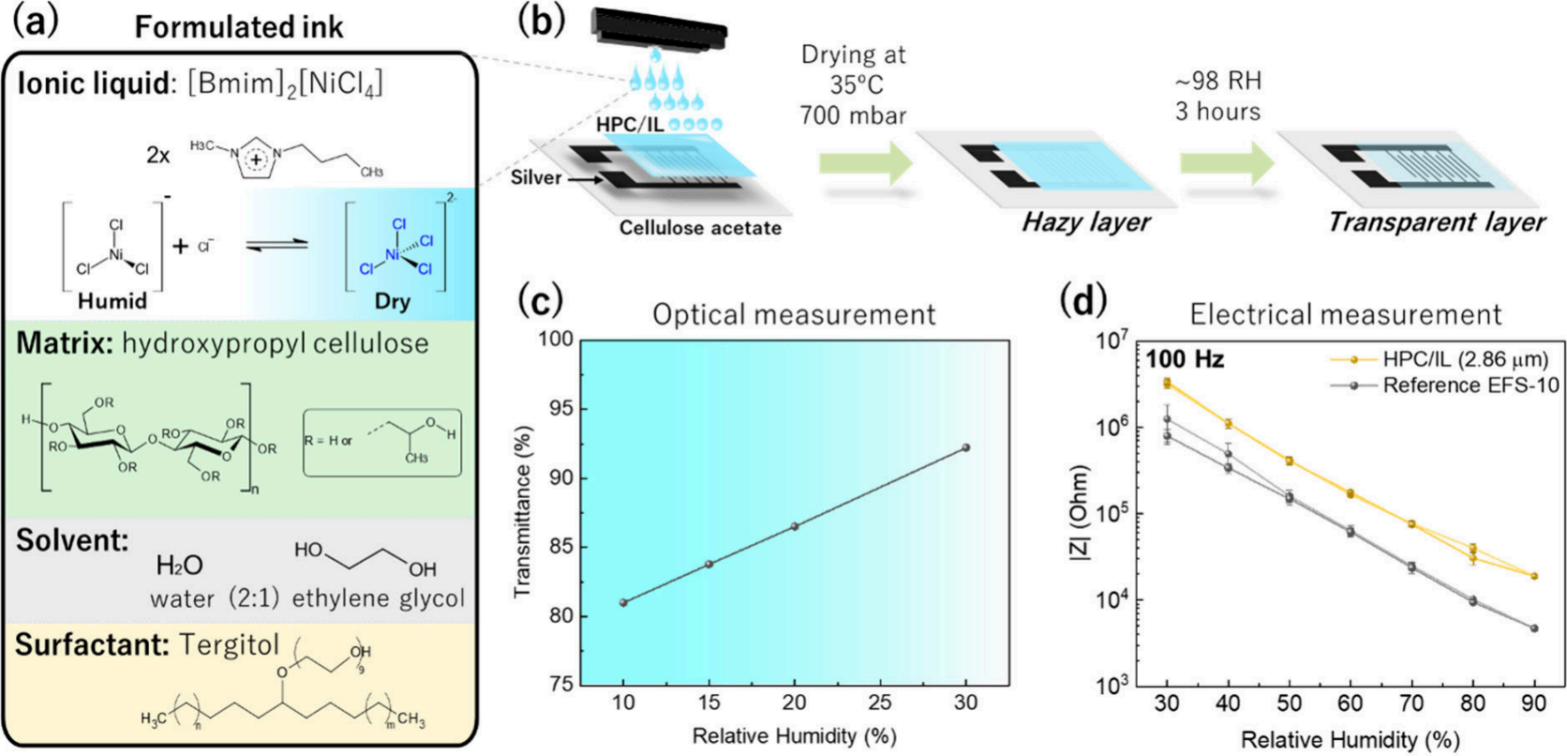
Representation of the formulation of the ink based on HPC/IL at 50 wt % (a). Scheme of printed layers, drying conditions, and humidity postprocessing conditions (b). Variation of the HPC/IL-CA sensor transmittance with the RH (10–30 RH%) at 708 nm (c) and impedance sensor variation as a function of the RH (30–90 RH%) of an active layer of 2.86 μm of thickness (d).

### 3.1 Ink Optimization and Performance

To formulate the IJP ink, it is necessary to adapt the viscosity (η) and surface tension (σ) to the specifications of the cartridge manufacturer, as summarized in Table 1. The ideal ink should present a viscosity of 4–8 mPa·s and a surface tension of 28–32 mN·m^–1^.^33^ On this basis, in the first printing tests, only water was used as the solvent; however, above 5 mg·mL^–1^ HPC, no droplet ejection from the nozzle was possible due to the high viscosity. At 5 mg·mL^–1^ of HPC, ejection of some drops was observed, but the ink was not able to jet reliably and stably because of the high surface tension (σ = 39.3 ± 0.1 mN·m^–1^). Apart from that, the mixture dries out in the outer part of the nozzles, leading to clogging and failed prints. Therefore, EG was included as a solvent in an H_2_O:EG volume ratio of 2:1. EG has been extensively used in industrial processes and is considered a “green” solvent. It is a nonvolatile and low-toxic solvent that can be synthesized from renewable biomass.^34,35^ The higher boiling point (197.3 °C) and good water miscibility of EG avoid the fast clogging of the printhead. As a consequence, the σ value was adjusted toward the ideal conditions of the cartridge, but the controlled droplet ejection was not yet achieved. Hence, 0.03 vol % of Tergitol was added as a biodegradable surfactant, which reduced the σ to 19.8 ± 2.5 mN/m facilitating the constant jetting of long-tailed drops. While the lowering of the σ to values lower than those recommended by the addition of a surfactant could produce the leaking of ink, no jetting was observed after switching off the nozzles. Finally, the optimized ink shown in Figure 1(a) was obtained by including 5 mg·mL^–1^ of IL (HPC/IL 50 wt %) in the mixture. It resulted in negligible changes in ink properties and remained printable without changing any printing condition. Therefore, the final composition of the ink results was HPC = 0.48 wt %, IL = 0.48 wt %, H_2_O = 63.72 wt %, EG = 32.84 wt %, and Tergitol = 0.03 wt %.

**Table 1:** Viscosity (η) and Surface Tension (σ) for an Ideal Ink Formulation for the Printheads and Solutions at 5 mg·mL^–1^ of HPC^a^

solution	η (mPa·s)	σ (mN·m^–1^)
ideal fluid	4.0–8.0	28.0–32.0
HPC + H_2_O	5.3 ± 0.1	39.3 ± 0.1
HPC + H_2_O:EG	9.2 ± 0.4	30.9 ± 1.2
HPC + H_2_O:EG + Tergitol	9.9 ± 0.4	19.8 ± 2.5
HPC/IL + H_2_O:EG + Tergitol	10.3 ± 0.4	20.5 ± 1.7

a Measured at a shear rate of 5000 s^–1^ and standard deviation given by the machine.

Figure 1(b) shows that the prints were dried in a vacuum oven at 35 °C and 700 mbar to accelerate the drying process. Higher temperatures were avoided as HPC exhibits a lower critical solution temperature (LCST) at 38–41 °C (1 atm), above which the cellulose derivative becomes insoluble in water. Similarly, the low pressure helps to accelerate the evaporation of EG and H_2_O without decreasing the LCST of HPC.^36,37^

The printed HPC/IL forms a hazy layer that does not allow us to observe the color change of the layer. Therefore, the prints were kept at 98 RH% for 3 h, where the humidity level was produced by a supersaturated solution of K_2_SO_4_ in a closed plastic box. Both HPC and the IL are highly hydrophilic, absorbing water molecules from the surrounding moisture, and the material slightly relaxes resulting in a homogeneous and transparent HPC/IL layer (Figure 1(b)). The change in the IL color, from colorless to cyan, is schematically explained in the top part of Figure 1(a). In the dehydrated state, it shows a cyan color as a result of the Ni­(II) tetrahedral structure ([NiCl_4_]^2–^). In the presence of water molecules, the Cl^–^ anions are replaced by the absorbed water molecules coordinated through a strong Ni^2+^–H_2_O bond which switches to a stable octahedral coordination sphere ([Ni­(H_2_O)_6_]^2+^) turning it colorless.^38^

The color change was monitored by UV–visible spectroscopy. Figure 1(c) shows the minimum transmittance values (at 708 nm) of an HPC/IL layer of 2.86 μm in the low humidity range of 10–30 RH%, where cyan color is visible. At 10 RH% the cyan color has the highest intensity, with a minimum transmittance of 81%. The color intensity decreases linearly with the humidity until 30 RH%, with a transmittance value of 92%; at a higher RH level, the sample remains colorless. Although the observable range of the cyan color is narrow, the range of the values falls into a characteristic limit for considering the high risk of potential wildfires, at which the use of machines that can sparkle or stubble burning should be avoided. Alternatively, it could serve as an indicator of the necessity to water the soil of crops in more precisely defined areas, helping to save water.

As a dual sensor, the electrical sensing properties were evaluated by measuring the impedance moduli (|*Z*|) at different frequencies. Unlike the optical properties, electrical measurements were collected within the consecutive range between 30–90 RH% at a constant temperature of 25 °C. Figure 1(d) summarizes the |*Z*| values at 100 Hz, which were recorded in steps of 10 RH%. Within this range, the |Z| values of the printed sensor decrease exponentially from 3 × 10^6^ Ω at 30 RH% to 2 × 10^4^ Ω at 90 RH%. Expressed in logarithmic scale, the sensor with 2.86 μm of HPC/IL presents a linear performance (*R* ^2^= 0.993) that is similar to the reference EFS-10 sensor, which presents more conductive behavior with |Z| decreased by half an order of magnitude. To quantitatively analyze the performance of the printed sensor, the sensitivity (*S*) of the sensor is described in eq 1. It represents the ratio between the maximum impedance change and its lowest value.

1 $$S=\frac{\Delta \left|Z\right|}{{\left|Z\right|}_{0}}=\frac{\left({\left|Z\right|}_{30RH\%}-|Z{|}_{90RH\%}\right)}{|Z{|}_{90RH\%}}$$

where Δ|*Z*| refers to the impedance difference between the extremes of the humidity range and |*Z*|_0_ refers to the impedance at 90 RH%. Both HPC/IL and reference humidity sensors demonstrate comparable sensitivity of 163 ± 10 and 161 ± 32, respectively. It is noteworthy that the sensor performs negligible hysteresis with the cycles, indicating good stability in all of the measured humidity ranges, while the commercial and our printed dual sensor present comparable surface area and similar performance. The sensor printed here on CA weighs 94 mg including the pins, almost half of the weight of the commercial sensor (170 mg). Hence, in terms of material efficiency, this printed sensor contributes to reducing the weight of global e-waste.

To study the stability of the printed sensor, it was exposed to a relative humidity higher than 90 RH% by introducing the sensor in a box with a supersaturated solution of KCl for 3.5 days as well as a reference sensor. Figure S1 shows the temperature and measured relative humidity for both sensors. The HPC/IL printed sensor demonstrated stability and high accuracy at this RH level based on the comparison with the reference sensor. In addition, the images of the electrodes in the inset demonstrate the stability of the silver IDEs in this system after continuous work at high humidity conditions.

To date, various humidity sensors based on ILs and their derivatives, such as poly­(ionic liquids) (PILs) and hyperbranched poly­(ionic liquid)­s (HPILs), have been reported. As indicated in Table 2, the HPC/IL sensor stands out in the high sensitivity for a wide humidity range and fast response and recovery time. The high sensitivity is ascribed to the water affinity of both the matrix (HPC) and the IL forming a homogeneous thin layer. Furthermore, the printed sensors performed small hysteresis of 1.5% RH, calculated as indicated in eq 2, where the maximum (RH_max_) and minimum RH (RH_min_) are referred to the values at the same RH level when cycling up and down humidity. The reference values (RH_ref_) correspond to the average at each RH level. For the calculation, the impedance values were transformed to RH% by using the logarithmic regression of the slope in Figure 1(d) [log_10_(*y*) = log_10_(0.918) + log_10_(3.4 × 10^7^)]. Besides, the response and recovery time of the printed sensors are comparable with those obtained for PIL, where the active layer is completely formed of polymerized ionic liquid. The response and recovery times were calculated as summarized in the Supporting Information and shown in Figure S2. The fast response of 0.8 s makes this sensor useful in applications where real-time monitoring is necessary as from breathing, heating, or human proximity.

$$hysteresis\;\left(\%\right)=\frac{\left|RH_{\max}-RH_{\min}\right|}{RH_{ref}}\times 100$$ 2

**Table 2:** Electrical Humidity Sensing Properties of HPC/IL Printed Sensor Compared with Reported Sensors Based on ILs

detection material	RH range	sensitivity	hysteresis	response/recovery time	ref
IL/MOF	5–30%	1.2	0.7 RH%	2.7/1.8 s	^39^
PIL	5–35%	48	0.2 RH%	1/10 s	^40^
HPIL	6–98%	∼32	6.0 RH%	4/107 s	^41^
[Bmim][Cl]	30–70%	3.4	–	140 s	^42^
PIL ([EMIM][TFSI])	10–80%	∼7	–	0.02 s	^43^
[Bmim]_2_[NiCl_4_]	30–90%	163	1.5 RH%	0.8/7.2 s	this work

On the other hand, the colorimetric or visual characteristics of the sensor have also been compared with other printed materials in Table 3. In general, the printed visual sensors are based on a color change produced by variation in the structural characteristics of the material with humidity. Contrary to other reported sensors, our sensor presents a color change at low RH levels, which makes it useful for indicating the limit for dry environmental conditions. On the other hand, these sensors have been extensively printed on nondegradable substrates, highlighting the suitability of our sensor in the development of “greener” electronics.

**Table 3:** Visual Humidity Sensing Properties of HPC/IL Printed Sensor Compared with Reported Printed Sensors

detection method	visual response	RH range	substrate	response time	reversibility	ref
CoCl_2_ color change	blue to pink	>60 RH%	3D-printed polylactic acid/Poly(ethylene oxide) (PLA/PEO)	<50 min	reversible	^44^
structural color change	structural colors in the visible range	–	nanolayered polymeric film	fast	reversible	^45^
conjugated polymer color change	blue to red	>80 RH%	polydiacetylene	<1 s	irreversible	^46^
structural color change	color changes across the visible spectrum	–	liquid crystal networks on glass and PDMS	–	reversible	^47^
structural color change	color shifts between blue, green, and red	0–100 RH%	CNC microfilms on PDMS and glass	<1 s	reversible	^48^
Ni-complex structure	cyan to transparent	<30 RH%	cellulose acetate	0.8 s	reversible	this work

### 3.2 Composite Material Characterization

Before optimization of the ink formulation toward inkjet printing, the optical, morphological, and chemical properties of HPC and [Bmim]_2_[NiCl_4_] were evaluated by analyzing blade-coated freestanding films at different IL concentrations. A maximum amount of 50 wt % of IL was successfully incorporated into HPC. Higher concentrations led to the IL being expelled from the matrix. Figure 2(a) visually compares the color of HPC/IL films under two conditions: humid (RT; 60 RH%) and completely dry (heated at 80 °C). A high transparency was observed when humid, a characteristic of HPC and hydrated IL. Then, a homogeneous cyan color was observed for the dried samples containing [Bmim]_2_[NiCl_4_], where the color saturation/intensity increased with the concentration.

**Figure 2:**
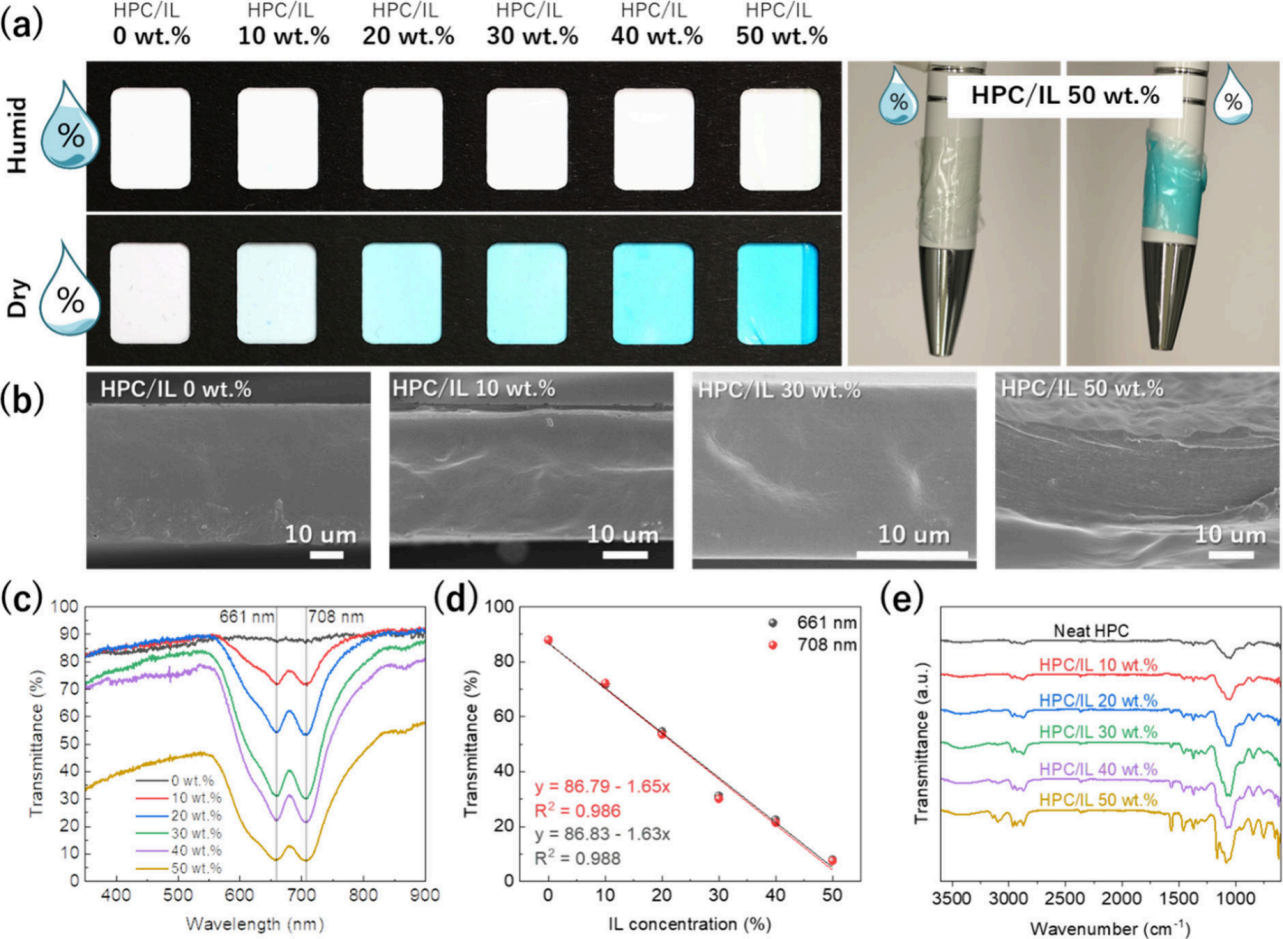
Optical images of HPC/IL composite films at different concentrations in humid and dry conditions and photographs of HPC/IL film at 50 wt % wrapping a pen in the humid and dry state (a). SEM images of the cross-section of the films (b), visible spectra in dry conditions (c), and representation of minimum transmittance peaks for the HPC incorporating different IL contents (λ = 661 and 708 nm) (d). FTIR spectra for neat HPC and HPC/IL composite films up to 50 wt % of IL (e).

It is remarkable that no phase separation of the IL inside the matrix can be observed in the cross-sectional SEM images of Figure 2(b). Reported polymers such as polyvinylidene fluoride (PVDF), poly­(l-lactic acid) (PLLA), and polyurethane acrylate (PUA), have poor miscibility with [Bmim]_2_[NiCl_4_] and thus tend to confine isolated nanopores of [Bmim]_2_[NiCl_4_] inside the matrix.^49–52^ The uniformity in our samples and the absence of phase separation suggest excellent physical compatibility between the IL and HPC even at the highest concentration. At HPC/IL 50 wt % a laminar-directed fracture is observed indicating the high ductility of the composite enhanced by the characteristic plasticizing effect of IL.^52^

The optical properties of the films at approximately 0 RH% (heated at 80 °C) are depicted in the visible spectra of Figure 2(c). Two absorption peaks were observed, one at 661 nm and the other at 708 nm, corresponding to the IL [NiCl_4_]^2–^ complex in its tetrahedral conformation. These wavelengths correspond to the red range of the visible spectrum giving the visible appearance is cyan color, a mixture of light green and blue. Figure 2(d) presents the transmittance percentages measured at both peaks, showing identical values for each sample. The transmittance intensity linearly decreases with the increasing IL concentration, where a transmittance of 8% for the film comprising 50 wt % of IL was observed. The linear proportionality between transmittance and IL concentration remarks the high homogeneity and good hosting capacity of the HPC.

FTIR spectroscopy was employed to analyze the chemical interactions between the IL and HPC. Figure 2(e) shows the FTIR spectra in the region from 3600 to 600 cm^–1^ for neat HPC and HPC/IL up to 50 wt %. As observed, the processing method does not induce chemical changes in HPC, as the main characteristic absorption bands remain present in the spectra. The wide absorption band centered at 3440 cm^–1^ is characteristic of the stretching of abundant O–H groups; narrower bands at 2972 and 2872 cm^–1^ correspond to C–H_2_ and C–H stretching, respectively. The group of bands centered between 1460–1265 cm^–1^ corresponds to C–H and O–H bending, while the pronounced band at 1050 cm^–1^ is assigned to C–O stretching vibration characteristic of carbohydrates. The absorption band at 837 cm^–1^ is attributed to C–O deformation and −CH_2_ rocking.^53^ The HPC absorption bands are not suppressed upon IL incorporation. The characteristic absorption bands of the IL are observed at 3139 and 3099 cm^–1^, corresponding to C–H stretching vibrations from the imidazolium ring of [Bmim]_2_[NiCl_4_]. The absorption bands at 2962, 2928, and 2887 cm^–1^, assigned to C–H, C–H_2_, and C–H_3_ stretching vibrations of lateral alkanes joined to the imidazolium ring, are overlapped with HPC bands. However, the peaks are better distinguished at higher IL concentrations. The narrow band at 1567 cm^–1^ belongs to the N–C–H rocking movement, while the bands around 1280 cm^–1^ are attributed to skeletal vibrations of the imidazolium ring. The absorption band at 1170 cm^–1^, more noticeable for the HPC sample with the highest IL content, 50 wt %, corresponds to the C–N stretching vibration of the ring. Finally, the absorption band at 753 cm^–1^ is attributed to the wagging of the N–C–H bonds in the imidazolium ring.^51,54–56^

### 3.3 Inkjet Printing Process

Before printing, the CA substrate was treated with Ar plasma for 5 min. It is known that plasma treatment cleans, activates, and cross-links the surface of polymers and thereby directly influences their surface-free energy.^57^ In this case, it can be observed in Figure S3 that plasma treatment enhanced the wettability of the HPC/IL-based ink, where the contact angle is reduced from 35 to 12 ± 4°.

After the drying process of HPC/IL 50 wt % at RT, the layers were hazy and white (Figure S4). The optical images of Figure 3 show the change from hazy (Figure 3(a)) to a completely colorless print after the treatment at ∼98 RH% for 3 h at RT (Figure 3(d)). In a closer look at the HPC/IL surface (without the silver electrode), the top SEM images demonstrate that the observed haziness was produced by a porous structure (Figure 3(b)). This porosity is likely associated with the faster evaporation of water compared with EG and the lower solubility of HPC in the remaining EG after the water is completely evaporated. The exposition of the prints to high humidity conditions slightly relaxed the sample, changing to a dense and colorless structure with a planar surface (Figure 3(e)). However, the high viscosity of HPC prevents the print from losing its pattern even when exposed to high humidity. Similarly, SEM-EDS analysis of HPC/IL layers printed on CA shows that the Ni element distribution maintains a uniform distribution in the printed layer before and after the humidity treatment, without any Ni-rich area, as shown in Figures 3(c) and 3(d), respectively. This result remarks the good compatibility between the IL and the matrix because the components are not separated or aggregated with the higher mobility produced after the water absorption.

**Figure 3:**
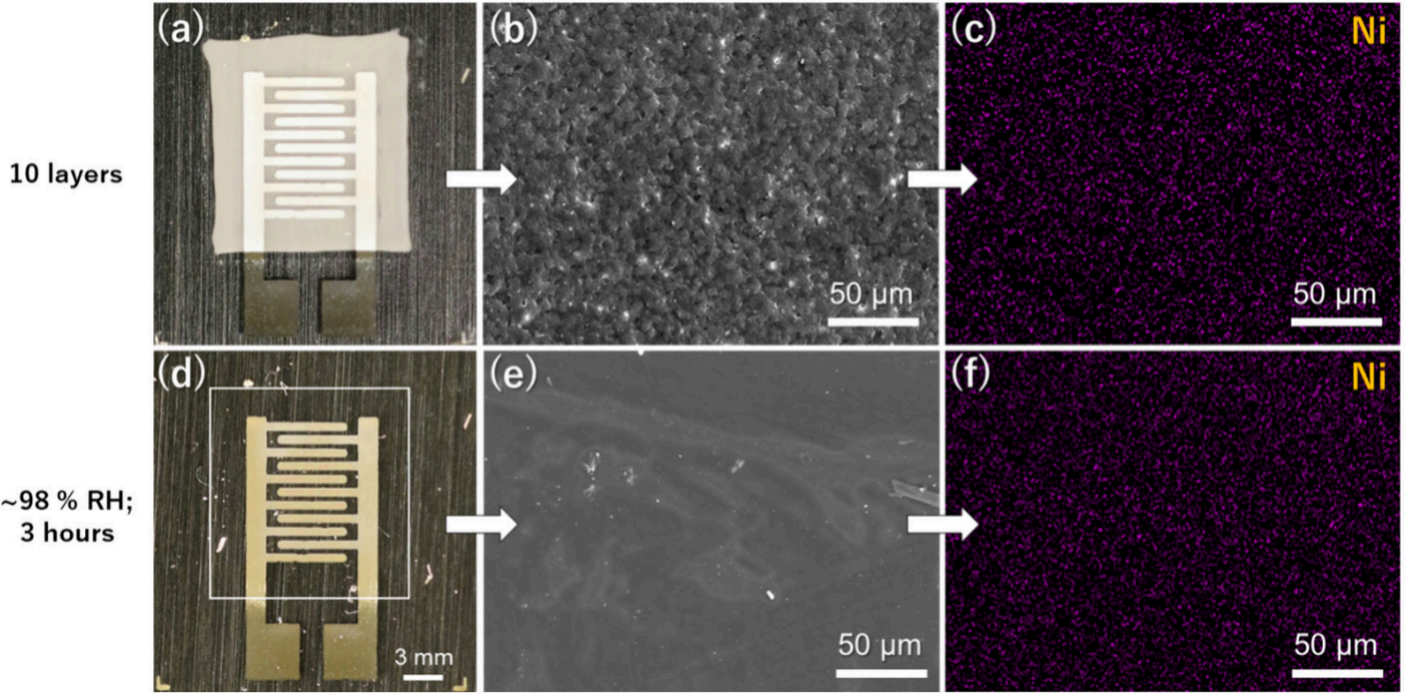
Photograph (a), SEM image (b), and SEM-EDX image for Ni element (c) of a print of HPC/IL 50 wt % with a thickness of 2.86 μm before the treatment at ∼98 RH% for 3 h. Photograph (d), SEM image (e), and SEM-EDX image for Ni element (f) of the respective print after treatment at high humidity conditions.

### 3.4 Sensor Characterization

Before ink development, it was necessary to prepare HPC/IL sensors at different IL concentrations to evaluate the electrical response. Solutions from pure HPC to HPC/IL 50 wt % were drop-cast on inkjet-printed IDEs on CA and evaluated by EIS in the 30–90 RH% range at 25 °C. The representative |*Z*| at different frequencies are represented in the Bode plot of Figure S6. At low frequencies, a plateau region is observed, while the impedance tends to decrease exponentially (linear in the “log vs log” scale) at high frequencies. This behavior can be modeled as an equivalent parallel RC circuit (Figure S5). Its impedance is described as follows:

3 $$Z\left(\omega \right)=\frac{1}{\frac{1}{R}+j\omega C}=\frac{R}{1+j\omega RC}$$

where *R* is the resistance, *C* is the capacitance, *j* is the imaginary unit (*j* ^2^ = −1), and ω represents the angular frequency (ω = 2π*f*). At high frequencies, the ionic response is insufficient to follow the rate of the alternating current (AC). Under these conditions, all of the current flows through the capacitor, effectively making a short circuit at infinite frequencies. Consequently, the impedance approaches zero when the capacitive path dominates. At low frequencies, the ions move following the electrical signal; therefore, all the current passes through the resistor, where the impedance in eq 3 is the same as the resistance because contains only the real part and the current is constant.^58^ The |*Z*| values in the plateau region decrease as the RH increases. As previously mentioned, the material absorbs water and the hydration enhances the mobility of the complexes when the chloride ions dissociate. The absorbed water further facilitates ion diffusion, thereby increasing the conductivity. Additionally, the strong capacity of HPC to form hydrogen bonds with water can detach certain chain segments, enabling structural rearrangements that open new pathways for ion mobility.^59^ It is noteworthy that even pure HPC presents a dynamic range at a very low frequency (0.1 Hz) for the different RH%, indicating its water absorption capacity. The increase of IL concentration introduces more charge carriers, which contribute to resistive behavior and water absorption capacity. As a consequence, the plateau region extends to higher frequencies and better conductivity. Therefore, the maximum plateau region encompasses frequencies from 0.1 to 100 Hz over the whole RH range for the sensor at 50 wt % of IL. However, the lowest frequencies are very slow for developing sensors to measure fast humidity changes.

We selected the highest frequency (100 Hz) to summarize the EIS performance of the drop-cast samples in Figure 4(a). At these conditions, only the sensors with 40 and 50 wt % of IL show impedance variations to develop a sensor in the measured relative humidity range. Given the similar behavior between the two compositions, 50 wt % IL (Figure 4(b)) was selected for printing to maximize the intensity of color in the printed sensors, where each printed layer is thinner than the ones obtained by drop casting. The error bars represent the standard deviation of three complete relative humidity cycles measured across three different sensors (*n* = 9). Hence, the maximum and minimum values of the standard deviation in the middle RH range (50 and 60 RH%) are transduced as an error of 1.2 RH%.

**Figure 4:**
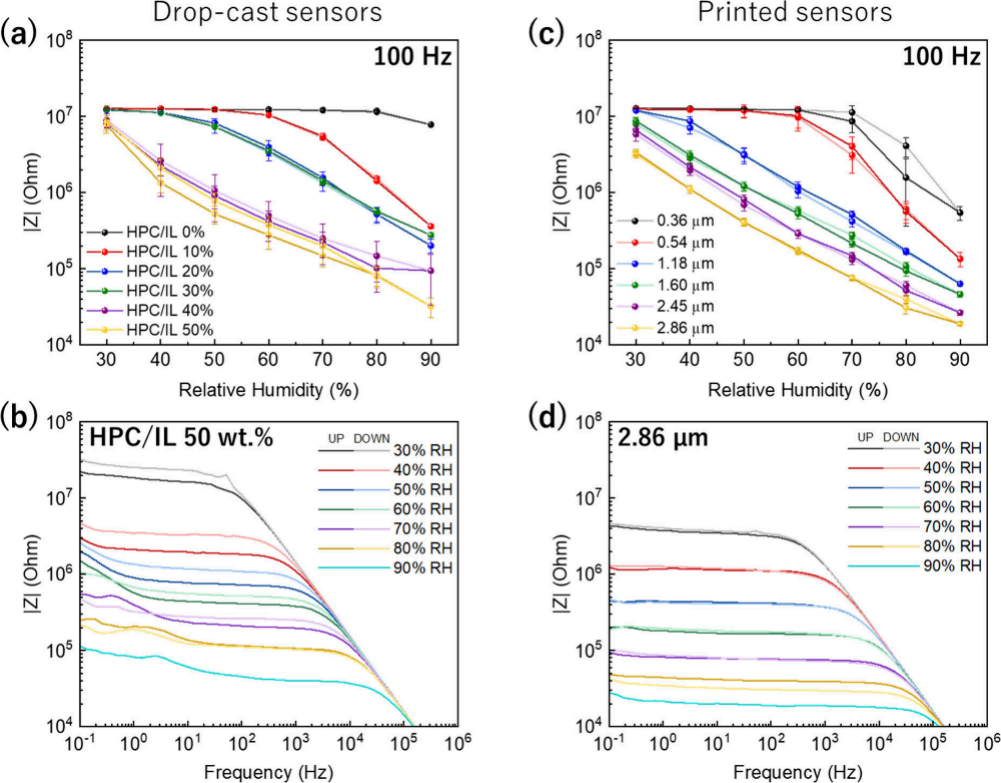
Impedance values at 100 Hz of ramping humidity cycles for HPC/IL drop-cast sensors at IL concentrations between 0 and 50 wt % of IL (a) and the Bode plot of HPC/IL drop-cast sensor at 50 wt % (b). Impedance values at 100 Hz of ramping humidity cycles for inkjet-printed sensors with HPC/IL at 50 wt % layer at different thicknesses (c) and Bode plot of the inkjet-printed sensor with an HPC/IL at 50 wt % and layer thickness of 2.86 μm (d).

In the printed samples, more than one layer was required to develop a functional sensor capable of covering the entire relative humidity range. A minimum of 1.18 ± 0.25 μm (four layers) was necessary to deposit enough material to achieve a linear response (log vs log) at 100 Hz, as shown in Figure 4(c). In this regard, thicker prints increase the number of ions available in the active area of the electrode digits. This results in a proportional reduction of the impedance modulus while maintaining the material’s sensitivity (with a consistent slope). These findings suggest that the print of a thickness of around 1.60 ± 0.11 μm (five layers) ensures the production of a humidity sensor that provides a reliable electrical signal corresponding accurately to the relative humidity level. However, a maximum thickness of 2.86 ± 0.29 μm (10 layers) of HPC/IL was printed to ensure the clear visibility of color change and reduce |*Z*| to values comparable to a commercial humidity sensor, as has been explained in Figures 1(c) and 1(d). The obtained thickness for 10 layers is still lower than inkjet-printed flexible and transparent humidity sensors based on polymers and carbon-based materials reported in the literature. Starke et al. printed 5 μm of humidity-sensitive polymeric particles on polyimide,^60^ and Ait-Mammar et al. printed 8 μm cellulose acetate butyrate on Kapton.^61^ On the other hand, thinner layers in the order of nanometers have also been printed on PET using grafted gold nanoparticles, which is a scarce element.^62^ In contrast, the sensor that we printed uses a biodegradable substrate, where the silver electrode could be recycled and the IL recovered by dissolving the active layer in water, as demonstrated in a previous publication.^63^ Furthermore, we used a low amount of active material with negligible waste due to the inkjet printing technique. The printed layer weighs 0.17 mg, where 0.085 mg corresponds to the IL.

The Bode plots of the drop-cast sensor containing 50 wt % of IL and the analogous printed sensor at a thickness of 2.86 ± 0.29 μm can be compared in Figures 4(b) and 4(d). The plateau region extends to higher frequencies (1000 Hz) with less hysteresis and higher conductivity in the printed sensors. One of the parameters that can produce the performance difference between the fabrication methods is the thickness of the active layer. Drop-cast sensors were 6 times thicker with a thickness of 17.6 ± 2.7 μm. The thicker and more irregular drop-cast layers absorb/desorb water at different rates, creating less efficient conductive pathways and reducing the conductivity. Further, some diffusion channels can be blocked or can locally trap water in the internal structure contributing to lower conductivity and hysteresis. In contrast, inkjet-printed samples present excellent IL distribution in uniform layers that can desorb water at the same rate for all surfaces and minimize water trapping.

Another key factor that can affect the sensor response is temperature. Normally, higher temperatures boost the ion mobility and increase conductivity. However, Figure S7 reveals a shift toward higher impedance when the temperature rises from 30 to 40 °C and also for higher temperatures. This drop in conductivity is directly related to the previously discussed LCST of HPC, which occurs between 38 and 41 °C. Beyond this temperature, HPC becomes insoluble in water. The literature suggests that this change marks a transition from a hydrophilic to a hydrophobic state, in which promoted self-bonding interactions form clusters.^64^ Therefore, the change in the affinity of polymer and water can obstruct pathways negatively affecting ion diffusion. This leads to two distinct regions: below and above the LCST. The impedance curves remain relatively consistent within their respective temperature ranges. However, for accurate record of RH values, the temperature should be simultaneously measured to adapt the calibration of this sensor.

Finally, the optical properties of the prints were analyzed. Figure 5(a) displays the visible spectra of the prints with increasing thickness by the addition of printed layers. The measurements were collected under dry conditions (heated to 80 °C). The cyan color starts to be noticeable for 1.18 μm and increases with every printed layer until the layer of 2.86 μm, showing a transmittance value of 81.7%. Using the latter sensor thickness, which demonstrated the highest electrical and optical response, we investigated how its color changes with varying humidity levels. Figure 5(b) shows the visible spectra measured in the climatic chamber at 40 °C for a print of 2.86 μm thickness, which is the lowest temperature in the climate chamber that permits the relative humidity to reach 10 RH%. At these conditions, 40 °C and 10 RH%, the print presents the cyan color as is observed in Figure 5(c). The transmission peaks exhibit a linear correlation with humidity, which corresponds to the results previously analyzed in Figure 1(c). In addition to the qualitative information of the print visualization, the proportional intensity of the color with the RH level allows the simple quantification of color, using a smartphone for the colorimetric analysis contributing to the point-of-need (PON) diagnostics.^65,66^

**Figure 5:**
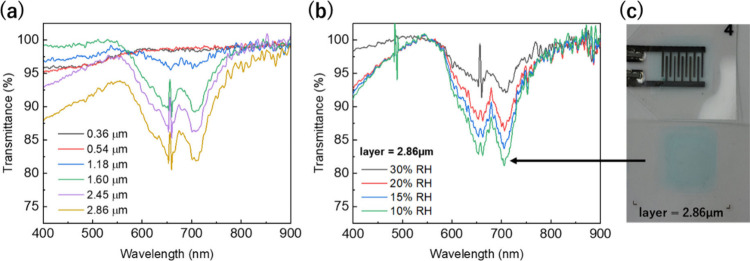
Visible spectra of HPC/IL with 50 wt % of IL printed with different thicknesses (a), visible spectra of sensor with a thickness of 2.86 μm between 30 and 10 RH% (b), and sample at 40 °C and approximately 10 RH% (c).

As 40 °C is close to the LCST, the effect of humidity on the transmittance was also analyzed across temperatures between 25 and 60 °C, as shown in Figure S8. At 30 RH%, transmittance values fluctuated randomly. Independently of the temperature, the printed layer absorbs some water that interacts with the nickel complexes. The random behavior could indicate that the composite material is close to the humidity level, where it fully maintains the octahedral [Ni­(H_2_O)_6_]^2+^ complex after the absorption of water. Subsequently, at 20, 15, and 10 RH%, the transmittance remained stable for the temperatures below and above the LCST.

As a proof of concept, the printed sensors were incorporated into a standard surgical mask and monitored by using an LCR meter at 100 Hz to evaluate their performance as breathing sensors. Within the mask, the relative humidity changes with inhalation and exhalation, as exhaled breath typically carries humidity levels between 41.9% and 91.0%, while inhaled breath lowers the RH levels.^67^ Figure 6 shows breath monitoring under various conditions, including rapid breathing, deep breathing, and speaking. The sensor effectively detects breath intensity and breathing speed, even at a rate of 1.8 min^–1^. Moreover, the sensor’s performance remains unaffected during speech, even when the exhalation rate becomes more irregular. The real-time demonstration of this capability can be seen in Supporting Video 1. This approach highlights how a simple, affordable, and personal facemask can be transformed into a functional breathing sensor.

**Figure 6:**
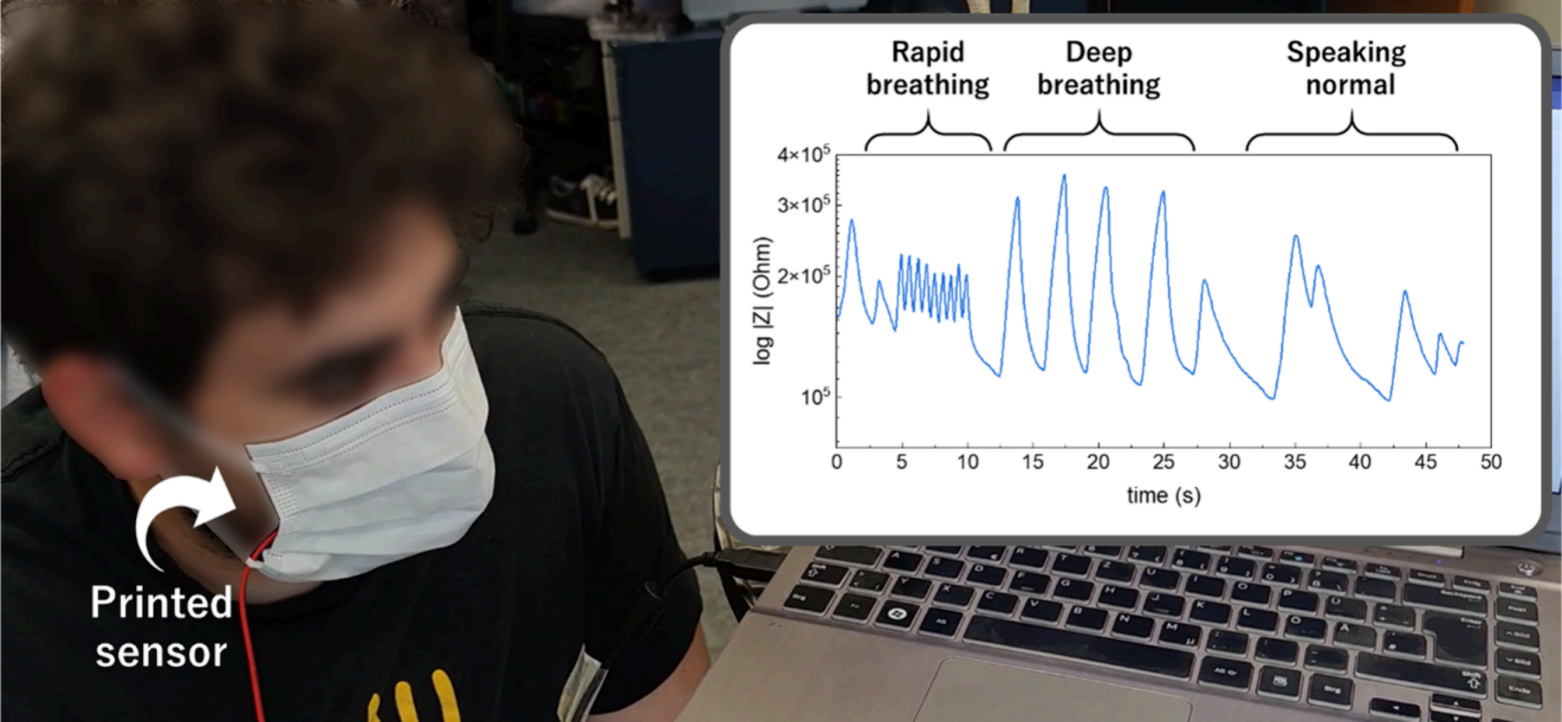
Breath monitoring was performed with a surgical mask and a printed sensor connected to an LCR meter at 100 Hz.

## 4 Conclusions

Here, we report the fabrication of an inkjet-printable, dual-response (electrical and visual) relative humidity sensor based on hydroxypropyl cellulose (HPC). This sensor uses an active layer consisting of HPC and ionic liquid (IL) [Bmim]_2_[NiCl_4_]. This IL has the ability to change the color from colorless in a humid state to cyan in the absence of water (thermochromism effect). Excellent physical compatibility between HPC and [Bmim]_2_[NiCl_4_] was observed, evidenced by the formation of homogeneous films without phase separation even at 50 wt % IL. The ink based on this composite material for the inkjet printing technique was formulated by adding ethylene glycol and water as solvent and Tergitol as a surfactant. The ink properties, such as viscosity and surface tension, were adjusted to align with the requirements of the printheads until reproducible printing was possible. A functional humidity sensor with dual-response was developed requiring 10 printed layers of the formulated ink, corresponding to a thickness of the active layer of 2.86 μm.

The EIS measurements in the range of 30–90 RH% at 100 Hz show that the |*Z*| values of the printed sensor decrease linearly (log vs log) from 3 × 10^6^ Ω (30 RH%) to 2 × 10^4^ Ω (90 RH%), comparable values with a commercial sensor. In addition, the sensitivity of 163 was more than three times higher compared with the literature, with very low hysteresis of 1.5% of RH and fast response and recovery times of 0.8 and 7.2 s, respectively. On the other hand, UV–vis characterization indicates that cyan color in printed sensors can be observed from humidities of 30 RH%, with a transmittance value of 92%, increasing the color intensity of the material with the RH level decrease until 10 RH% with a transmittance of 81%.

Therefore, the sensor offers the potential for a variety of applications. The electrical response makes it suitable for applications including comfort and healthcare monitoring, proximity sensing, the food industry, and agriculture. The visual response can serve as a quick indicator of extreme wildfire risk, the need for irrigation in smart agriculture, or the integrity of dehydrated food packaging. Overall, the potential of these sustainable materials and printing technologies has been demonstrated for the development of low-cost, high-performance sensors for various applications.

## Supplementary Material





## Notes

Dynamic characterization, temperature, and long-term experiments with the details on data analysis for EIS, Bode plots for drop-cast sensors, microscope images, and photographs of layers and sensors, and UV–vis spectra of sensors at different RH% (PDF)
Real-time demonstration of the sensor effectively detecting breath intensity and breathing speed (MP4)
