# Supplementary material for: Sustainable Fully Inkjet-Printed Humidity Sensor Based on Ionic Liquid and Hydroxypropyl Cellulose
Source: ACS Appl Mater Interfaces. 2025 May 25;17(22):32680–90. doi: 10.1021/acsami.5c00505 (PMC12147771; doi:10.1021/acsami.5c00505)
Supplement: Supplementary file 1 [file am5c00505_si_001.pdf]

SUPPORTING INFORMATION

Sustainable Fully Inkjet-Printed Humidity Sensor Based on Ionic Liquid and Hydroxypropyl Cellulose

*Mikel Rincón-Iglesias[1,2,3], Peter Krebsbach[2,3], Daniela M. Correia[4], Cristian Mendes-Felipe[1], Senentxu Lanceros-Méndez[1,5], Gerardo Hernandez-Sosa[2,3,6]**

[1]BCMaterials, Basque Center for Materials, Bldg. Martina Casiano, UPV/EHU Science Park Barrio Sarriena s/n, 48940 Leioa, Spain.

[2]Light Technology Institute, Karlsruhe Institute of Technology, Engesserstr. 13, 76131 Karlsruhe, Germany.

[3]InnovationLab, Speyerer Straße 4, 69115 Heidelberg, Germany.

[4]Centre of Chemistry, University of Minho, Braga, 4710-057 Portugal

[5]IKERBASQUE, Basque Foundation for Science Plaza Euskadi 5, Bilbao 48009, Spain.

[6]Institute of Microstructure Technology, Karlsruhe Institute of Technology, 76344 Eggenstein-Leopoldshafen, Germany.

*Email: gerardo.sosa@kit.edu

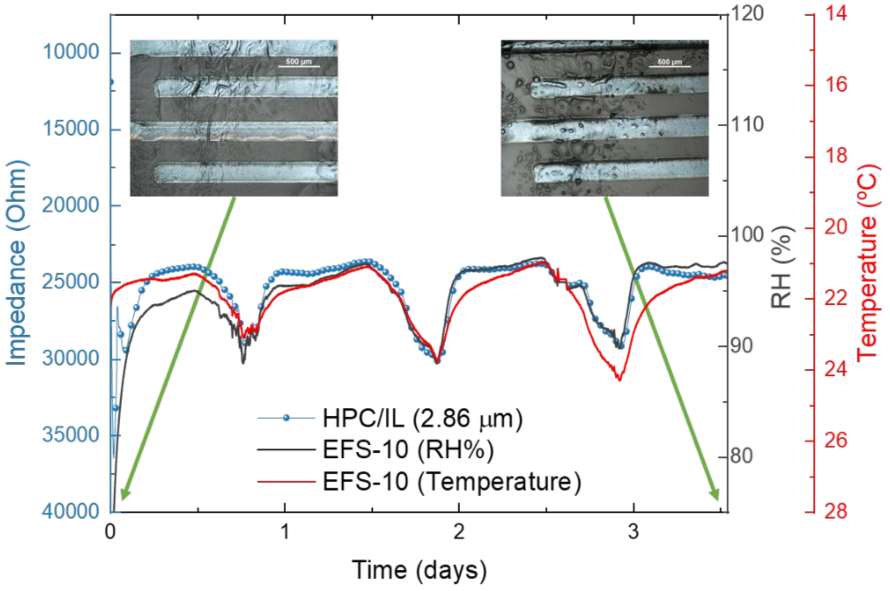

Figure S1. Measurement of the stability of the printed sensor at RH higher than 90 RH% and compared with the reference sensor, which measured RH and temperature. The insets show the electrodes with the HPC/IL printed layers on top before (left) and after (right) the measurement.

Long-term Measurement:

For the long-term measurement, the sensor was placed inside a PMMA box (7x10x13cm) with electrical leads connected to an LCR meter (Sourcetronic ST2822E, connected to Python, readout every 10s) and a supersaturated KCl solution. For comparison, a reference sensor HYT271 (IST AG) recorded the RH and temperature during the experiment inside the box via an Arduino Uno

and Phyton script (readout every 1s). The experiment was conducted in an office room with changing temperatures due to the heating cycles during day and night as recorded by the reference.

Response and Recovery Time:

The custom-made setup to determine the detection and recovery speed of the sensors is described in a previous paper. [62] In short, dry and wet N2-gas flows were alternatively directed on the sensors in a 6x6x6cm³ box. Wet N2 was generated by bubbling the gas through a water bottle. The impedance was recorded with an LCR meter (Sourcetronic ST2832) as shown in **Figure S2**.

The response and recovery speed of the sensors were calculated by determining the time in which 90% of the impedance change Δ|Z| was completed. For that, an average of the values during the plateau of dry gas streams was determined and compared to the minimum value of the wet phase to determine Δ|Z|. The response and recovery times were then calculated from the last data point before the change and the first data point that fulfills 0.9*Δ|Z|.

A mean of four such cycles gives the following values: t_{90}, response = 0.8 ± 0.1 s, and t_{90}, recovery = 7.2 ± 0.6 s.

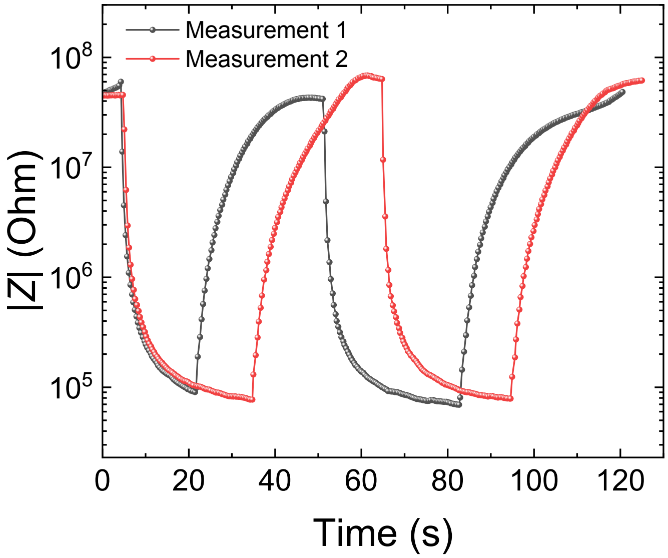

Figure S2: Two impedance recordings of the humidity sensor during dry and wet gas stream cycling to determine the detection and recovery speed.

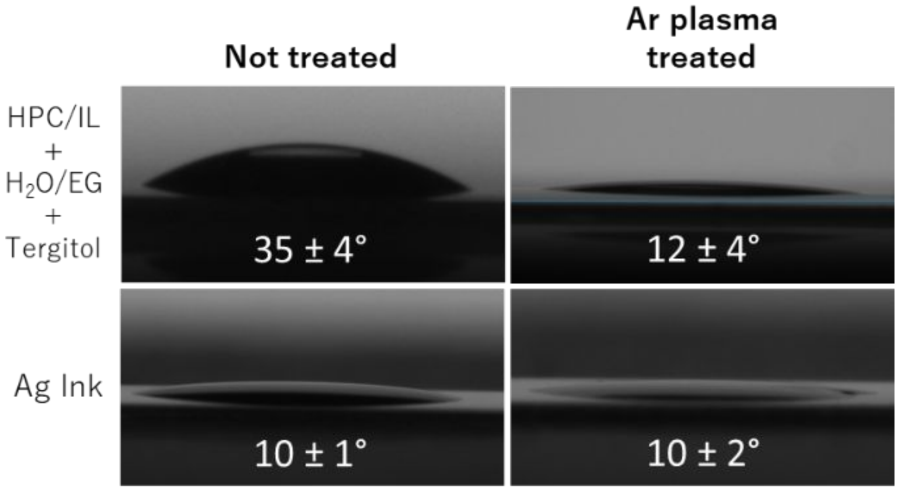

Figure S3. The contact angle of the formulated HPC/IL-based ink and Ag ink before (left) and after (right) an argon plasma treatment of the substrate, CA.

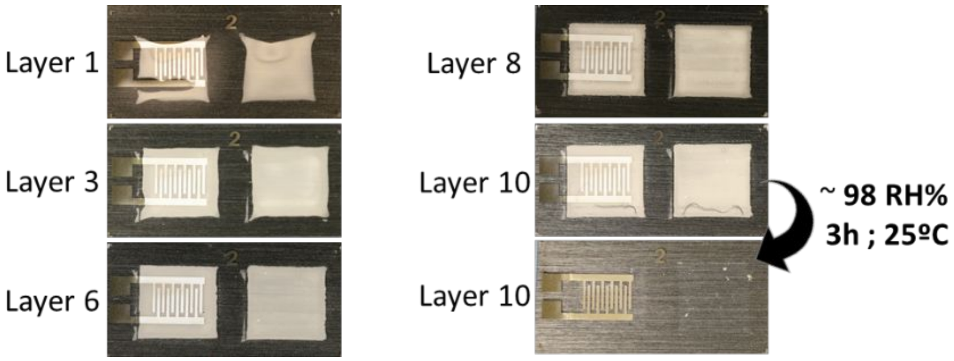

Figure S4. Dry HPC/IL printed layers on Ag silver IDEs and on pure CA substrate before humidity treatment. The bottom right photograph shows 10 HPC/IL printed layers after humidity treatment at ~98 RH% for 3 hours at RT.

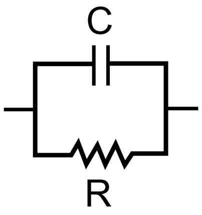

Figure S5. Scheme of an equivalent parallel RC circuit.

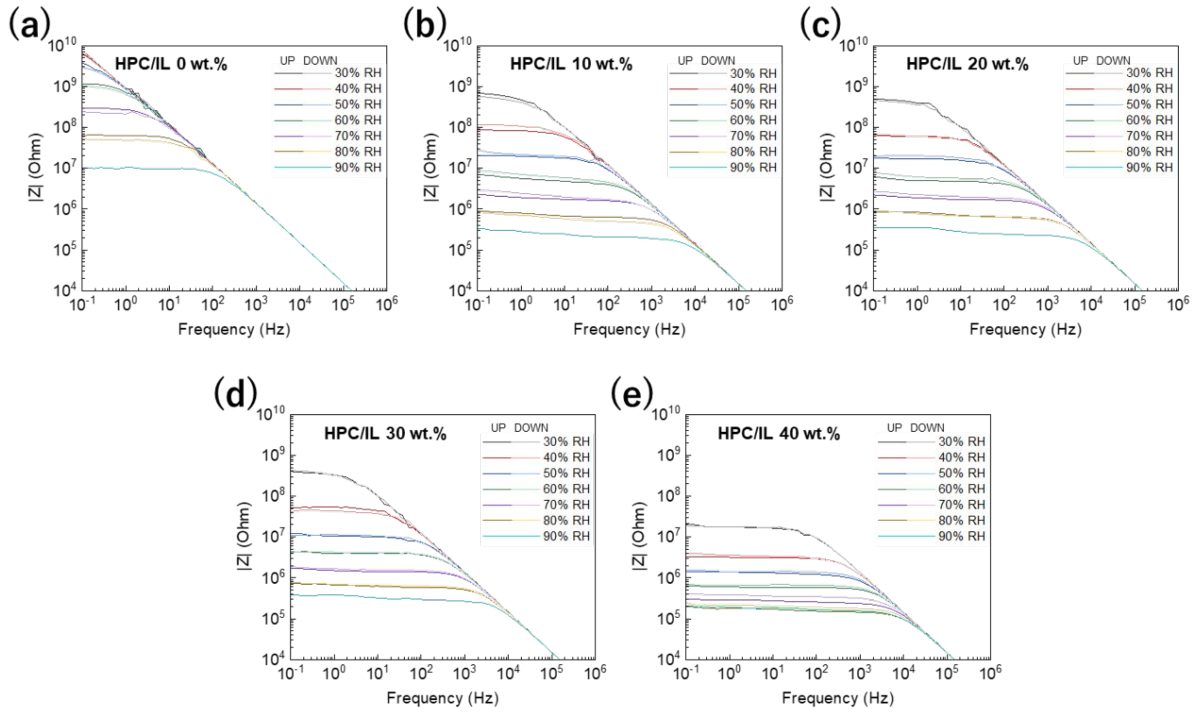

Figure S6. Bode plots for drop-cast samples of HPC/IL at (a) 0, (b)10, (c)20, (d)30, and (e)40 wt.% while ramping humidity cycles.

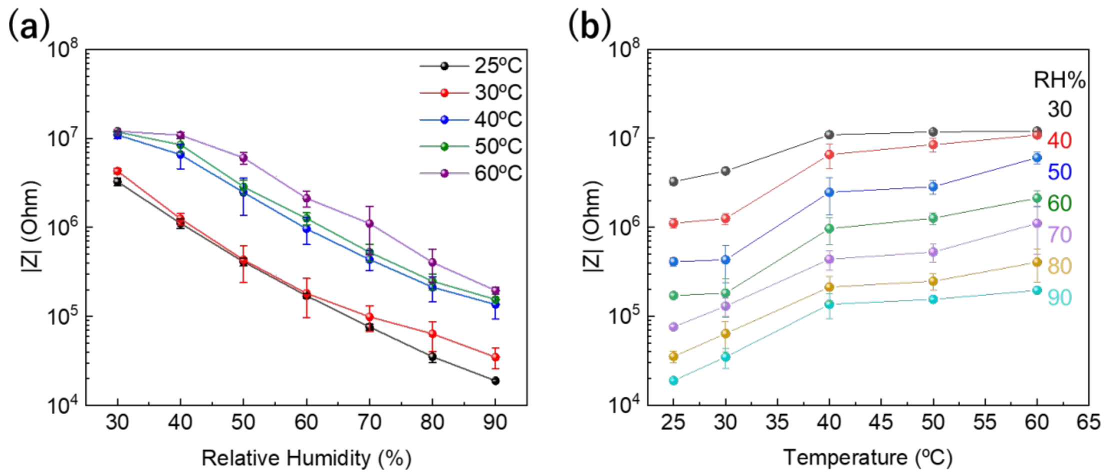

Figure S7. (a) Effect of temperature on the humidity response of inkjet-printed samples containing 50 wt.% IL at 100 Hz. (b) Representation of RH% response variation at temperatures in the range of 25-60°C.

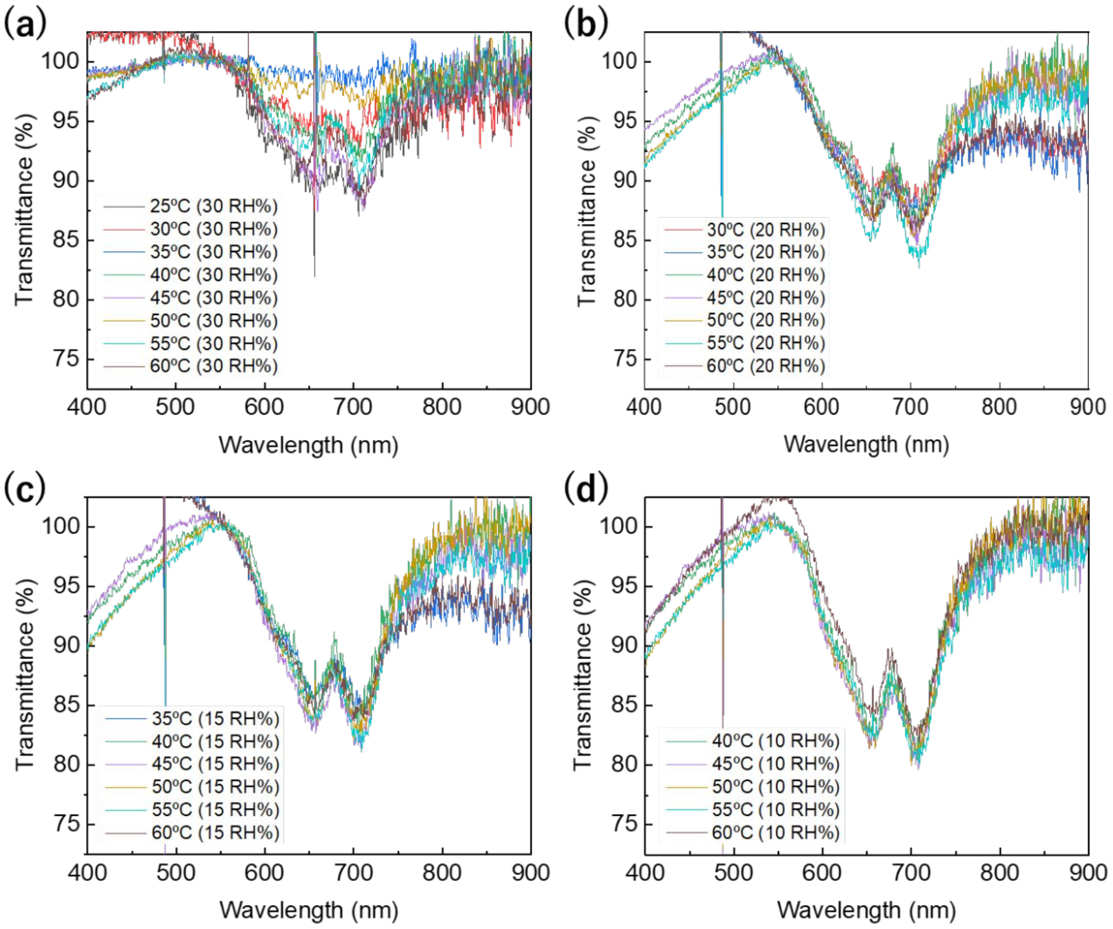

Figure S8. UV-vis spectra of the printed sensor with a thickness of 2.86 μm at different temperatures for 30 RH% (a), 20 RH% (b), 15 RH% (c), and 10 RH% (d).
